# Induced pluripotent stem cell derived cardiomyocytes as models for cardiac arrhythmias

**DOI:** 10.3389/fphys.2012.00346

**Published:** 2012-08-31

**Authors:** Maaike Hoekstra, Christine L. Mummery, Arthur A. M. Wilde, Connie R. Bezzina, Arie O. Verkerk

**Affiliations:** ^1^Department of Clinical and Experimental Cardiology, Heart Failure Research Center, Academic Medical Center, University of AmsterdamAmsterdam, Netherlands; ^2^Department of Anatomy and Embryology, Leiden University Medical CenterLeiden, Netherlands; ^3^Department of Anatomy, Embryology, and Physiology, Heart Failure Research Center, Academic Medical Center, University of AmsterdamAmsterdam, Netherlands

**Keywords:** induced pluripotent stem cells, cardiac arrhythmia syndromes, electrophysiology, cardiomyocytes, human, iPS, heart

## Abstract

Cardiac arrhythmias are a major cause of morbidity and mortality. In younger patients, the majority of sudden cardiac deaths have an underlying Mendelian genetic cause. Over the last 15 years, enormous progress has been made in identifying the distinct clinical phenotypes and in studying the basic cellular and genetic mechanisms associated with the primary Mendelian (monogenic) arrhythmia syndromes. Investigation of the electrophysiological consequences of an ion channel mutation is ideally done in the native cardiomyocyte (CM) environment. However, the majority of such studies so far have relied on heterologous expression systems in which single ion channel genes are expressed in non-cardiac cells. In some cases, transgenic mouse models have been generated, but these also have significant shortcomings, primarily related to species differences. The discovery that somatic cells can be reprogrammed to pluripotency as induced pluripotent stem cells (iPSC) has generated much interest since it presents an opportunity to generate patient- and disease-specific cell lines from which normal and diseased human CMs can be obtained These genetically diverse human model systems can be studied *in vitro* and used to decipher mechanisms of disease and identify strategies and reagents for new therapies. Here, we review the present state of the art with respect to cardiac disease models already generated using IPSC technology and which have been (partially) characterized. Human iPSC (hiPSC) models have been described for the cardiac arrhythmia syndromes, including LQT1, LQT2, LQT3-Brugada Syndrome, LQT8/Timothy syndrome and catecholaminergic polymorphic ventricular tachycardia (CPVT). In most cases, the hiPSC-derived cardiomyoctes recapitulate the disease phenotype and have already provided opportunities for novel insight into cardiac pathophysiology. It is expected that the lines will be useful in the development of pharmacological agents for the management of these disorders.

## Introduction

Cardiac arrhythmias can be life threatening and are a major cause of morbidity and mortality in developed nations (Wolf and Berul, 2008). In older patients, most arrhythmic sudden deaths occur in the setting of acute ischemia or coronary artery diseases (Zipes and Wellens, 1998). In younger patients, the great majority of sudden arrhythmic deaths have an underlying genetic cause (Wilde and Bezzina, 2005). These can be broadly subdivided into those associated with structural heart disease (such as hypertrophic cardiomyopathy) and those associated with electrical disease in the structurally normal heart (Wolf and Berul, 2008).

Over the last 15 years, much progress has been made in identifying the clinical phenotypes and cellular and genetic mechanisms underlying the various primary Mendelian arrhythmia syndromes, including the Long QT syndrome (LQTS), Brugada Syndrome (BrS), and catecholaminergic polymorphic ventricular tachycardia (CPVT) (Wilde and Bezzina, 2005). This has provided important insights into these disorders and as a consequence improved the management of affected patients. The availability of genetic tests has added an important diagnostic tool permitting early (presymptomatic) identification of patients at risk and allowing for the timely implementation of preventive strategies (Hofman et al., 2010). Studies into genotype–phenotype relationships have uncovered important gene-specific aspects of disease and indicated that patient management must take the nature of the gene affected into consideration (Priori, 2004). However, there is considerable variation in phenotypic expression of arrhythmia syndromes even within families carrying the same mutation (Scicluna et al., 2008).

Studying the electrophysiological and molecular consequences of a mutation associated with cardiac arrhythmia is ideally done in the native cardiomyocyte (CM) environment. However, obtaining ventricular cardiac biopsies from patients is a highly invasive procedure and not without significant risk. Consequently the majority of functional studies on specific mutations associated with the Mendelian rhythm disorders have relied on heterologous expression systems, primarily *Xenopus oocytes*, human embryonic kidney (HEK) cells, and Chinese Hamster Ovary (CHO) cells (Watanabe et al., 2008), in which the mutated ion channel of interest is expressed. Such cellular models have significant shortcomings since they lack important constituents of cardiac ion channel macromolecular complexes that might be required to reproduce the exact molecular and electrophysiological phenotype of the mutation. For example, the behavior of the Na^+^ channel in cell expression systems seems to be different from that in CMs (Remme et al., 2008). One way of overcoming this has been to generate transgenic mice carrying specific mutations (Sabir et al., 2008). However, the generation of such mouse models is costly and time-consuming and not practical for high-throughput screening of rare inherited arrhythmia mutations. Moreover, there remain crucial differences between mouse and human cardiac electrophysiological characteristics, such as the high basal heart rate (>500 bpm), the very negative action potential (AP) plateau phase, and the short AP compared of the mouse compared to human (Watanabe et al., 2011). These differences are amongst others due to the different biophysical properties in the transient outward currents (*I*_to_) of human and mouse (for review, see Nerbonne and Kass, 2005).

The discovery of somatic cell reprogramming to generate induced pluripotent stem cells (iPSC) (Takahashi and Yamanaka, 2006) has created much excitement because of the possibility of producing unique patient- and disease-specific human iPSC (hiPSC) lines (Takahashi and Yamanaka, 2006; Takahashi et al., 2007; Yu et al., 2007). With this technique, somatic cells can be turned into embryonic stem cell-like cells which can differentiate into all cells of the human body and be propagated indefinitely in culture. Thus, hiPSC can provide investigators with genetically diverse human model systems to study mechanisms of disease and identify strategies for potential new therapies. Zhang et al. (2009) were the first to show that hiPSC can differentiate to functional CMs, making it possible to generate patient-specific human CMs which are by definition on different genetic backgrounds. hiPSC-derived CMs (hiPSC-CMs) therefore represent a new model system for studying Mendelian arrhythmia syndromes.

Here we provide a short overview of hiPSC generation, culturing, and differentiation methods. Further we will discuss in detail the electrophysiological characteristics of hiPSC-CMs and review hiPSC models for cardiovascular diseases, including LQT1, LQT2, LQT3/BrS, LQT8/Timothy syndrome, and CPVT.

## Derivation of hiPSC models

### Cell origin

Although the first hiPSC lines were derived from dermal fibroblasts (Takahashi et al., 2007) hiPSC can now be generated from a wide variety of somatic cells. It is important to consider easily accessible sources, which are efficient to reprogram and give minimal burden to the patient. Easily accessible sources used successfully for reprogramming include keratinocytes from skin or plucked hair (Aasen et al., 2008), peripheral blood (Loh et al., 2009), mesenchymal cells in fat (Sun et al., 2009), dental pulp (Tamaoki et al., 2010), and oral mucosa (Miyoshi et al., 2010).

### Cell reprogramming

Somatic cells can be reprogrammed to a pluripotent state by introducing pluripotency-associated genes. The first iPSC reported were generated by transducing mouse fibroblasts with four retroviral vectors OCT4, SOX2, KLF4, and C-MYC (Takahashi and Yamanaka, 2006). The first hiPSC were generated using the same four retroviral vectors (Takahashi et al., 2007) or OCT4, SOX2, LIN28, and NANOG (Yu et al., 2007). Later studies reported reprogramming with other combinations and numbers of pluripotency factors. The hiPSC thus generated can be kept in culture indefinitely using a variety of undefined fibroblast feeder cells and fetal calf serum-based methods (Takahashi et al., 2007; Yu et al., 2007) or defined mTESr/Matrigel-based protocols. Transplantation of hiPSC into immune-compromised mice leads to the formation of teratomas with derivatives of the three embryonic germ layers, demonstrating the pluripotent potential of these cells (Takahashi et al., 2007; Yu et al., 2007). In addition, differentiation of hiPSC *in vitro* also results in derivatives of the three germ layers. The review by Narsinh et al. (2011) provides a detailed overview of the methods to reprogram somatic cells to iPSC and discusses the advantages and disadvantages of the different techniques.

### Generation of iPSC-derived cardiomyocytes

When iPSC are removed from differentiation suppression conditions and/or when grown in suspension aggregates [called embryoid bodies (EBs)] spontaneous differentiation to cells of the three germ layers occurs. CMs originate from the mesodermal germ layer, so that CM differentiation first requires efficient differentiation toward the mesodermal lineage. Directed differentiation toward the cardiac lineage is mainly achieved by one of the following strategies: (1) The first involves the formation of EBs in the presence of growth factors and repressors known to influence heart development (Kehat et al., 2001); (2) The second relies on the influence of endoderm on cardiac differentiation during embryogenesis (Mummery et al., 2003). Here, co-culture of iPSC with mouse END-2 is used to produce CMs; (3) The third involves monolayer culture at high density of iPSC seeded on Matrigel with sequential treatment with activin A and BMP4 (Laflamme et al., 2007). This method was developed using human embryonic stem cells (hESC) but has been transferred to hiPSC. Beating areas from differentiated EBs usually appear in 7–10 days. These EBs can be microscopically dissected and dissociated in single cells. For electrophysiological and immunofluorescence analysis, the dissociated cells can be seeded onto glass coverslips.

## Characteristics of hiPSC-derived cardiomyocytes

### Molecular and structural characteristics

The first hiPS-CMs were generated by Zhang et al. (2009). In these cells the investigators examined the gene expression of the transcription factor Nkx2.5, the myofilament proteins cardiac troponin T, α-myosin heavy chain, α-actinin, the atrial and ventricular isoforms of myosin light chain 2, atrial natriuretic factor, and phospholamban (PLN). Low levels of cardiac troponin T and the atrial isoform of myosin light chain 2 were found in undifferentiated hiPSC and high expression of all the cardiac genes were found in the hiPSC-CMs, which was comparable to the expression of these genes in adult ventricular myocardium. Immunohistochemistry showed a typically striated pattern for α-actinin and myosin light chain. However, these cells had multi-angular morphologies and relatively disorganized sacromeres (Dick et al., 2010). Novak and co-workers demonstrated by transmission electron microscopy analysis that hiPSC-CM had an immature ultra structure without t-tubuli (Novak et al., 2012).

### Action potentials

Using the patch clamp technique, Zhang et al. (2009) were the first to measure the APs in spontaneously contracting cells isolated from hiPSC-EBs. The majority of the cells showed ventricular-like APs (70–74% of cells for two distinct hiPSC lines), but atrial-like and nodal-like APs were also observed. The distinction was made on AP phenotype, with a negative diastolic membrane potential, a rapid AP upstroke and a long plateau phase for ventricular-like APs. The absence of a prominent plateau phase was a characteristic of atrial-like APs, resulting in shorter AP duration compared to ventricular-like APs. Nodal-like APs showed a more positive maximum diastolic potential (MDP), a slower AP upstroke and a prominent phase 4 depolarization. Other studies also described ventricular-like, atrial-like, and sometimes nodal-like APs (Moretti et al., 2010; Fatima et al., 2011; Itzhaki et al., 2011a; Ma et al., 2011; Matsa et al., 2011; Jung et al., 2012; Lahti et al., 2012), with the ventricular-like phenotype being the most prominent AP form (76–48%) (Zhang et al., 2009; Moretti et al., 2010; Itzhaki et al., 2011a; Ma et al., 2011; Lahti et al., 2012). Comparison of hiPSC-CMs to hESC-CMs seems valuable, since hESC-CMs are more established. In hESC-CM, ventricular-like APs are also observed more frequently [50–60% (Zhang et al., 2009, 2011)]. Moretti et al. (2010) employed single-cell reverse-transcriptase-PCR in combination with patch-clamp in the same cell to show that the designation as ventricular-like, atrial-like, and nodal-like APs based on cellular electrophysiological features correlated with gene-expression of specific myocyte-lineage markers.

Table 1 summarizes the reported AP characteristics of hiPSC-CMs, hESC-CMs, and native ventricular CMs. The APs measured in hiPSC-CM differ from APs measured in freshly isolated native CMs (Table 1). The first remarkable difference is that most of the hiPSC-CMs studied, including the ventricular-like and atrial-like cells, are spontaneously active, with beating rates between 28 and 108 bpm (Table 1). Whether, in these studies, the spontaneous activity was used as a tool for CM selection and that non-beating CMs were also present, or whether it is a typical feature of hiPSC-CMs is unknown. We recently (Davis et al., 2012) performed experiments on non-spontaneously beating hiPSC-CM. For this we selected quiescent cells which were able to contract upon field stimulation. In these non-spontaneous beating hiPSC-CM, the resting membrane potential (RMP) was more negative then the MDP in most studies reporting on spontaneously active hiPSC-CMs (Table 1). Compared with native human ventricular CMs, where the reported RMP varies from –81.8 to –87 mV (Table 1), the MDP of ventricular-like hiPSC-CM APs is less negative with values ranging from –57 to –75 mV (Table 1). In spontaneously beating hiPSC-CM, the ventricular-like AP has a maximal upstroke velocity (*dV*/*dt*_max_) ranging from 9 to 40 V/s which is slow compared to those non-spontaneously beating hiPSC-CM with a *dV*/*dt*_max_ of 115 V/s, and native ventricular CMs with a *dV*/*dt*_max_ of 215–234 V/s (Magyar et al., 2000). The duration of ventricular-like hiPSC-CM APs, for example at 90% of repolarization (APD_90_), is longer in spontaneous active cells (313–495 ms) compared to non-spontaneously active hiPSC-CMs (173 ms) and native freshly isolated CMs (213–351 ms) (Table 1). The AP amplitude (APA) for most ventricular-like hiPSC-CM APs (87–113 mV) is comparable to native ventricular CMs (104–106 mV), which due to the depolarized MDP in hiPSC-CM results in a higher overshoot of the hiPSC-CM AP. The direct comparison between the APs is complicated by the differences in experimental techniques used. Most hiPSC-CM studies used a temperature between 35 and 37°C; only in the study of Itzhaki was a temperature of 32°C used. This could be a possible explanation of the depolarized MDP, slow *dV*/*dt*_max_ and long APD_90_ in this latter study (Itzhaki et al., 2011a). The majority of the studies used the ruptured whole-cell patch-clamp technique, and three studies (Ma et al., 2011; Davis et al., 2012; Lahti et al., 2012) used the perforated patch-clamp technique. In the latter technique it is possible to perform experiments more close to physiological conditions since cell dialysis is minimal and EGTA, a buffer for Ca^2+^ ions, is absent.

**Table 1:** **Action potential characteristics in hiPSC-CM and human ventricular cardiomyocytes**.

**Cell type**	**BPM**	**APD_90_ (ms)**	**dv/dt_max_ (V/s)**	**APA (mV)**	**MDP/RMP (mV)**	**Exp. conditions**	**References**
iPSC-CM V IMR90C4	43.8 ± 2.7	320.1 ± 17	40.5 ± 4.6	87.7 ± 2.6	−63.5 ± 1.7	37°C, me LJP: corr.	Zhang et al., 2009
iPSC-CM V ForeskinC1	44.2 ± 3.5	312.5 ± 11.2	27.2 ± 3.7	87.9 ± 2.4	−63.3 ± 1.5	37°C, me LJP: corr.	Zhang et al., 2009
iPSC-CM V	68.2 ± 2.7	381.3 ± 35.3	9 ± 0.2	107.8 ± 2.1	−63.5 ± 2.1	35°C, wc LJP:nk	Moretti et al., 2010
iPSC-CM V	72 ± 1.2	314.4 ± 17.6	26.8 ± 6.3	113.2 ± 2.4	−63.4 ± 1.3	36°C, pp LJP: nk	Lahti et al., 2012
iPSC-CM V	35.5 ± 2.1	414.7 ± 21.8	27.8 ± 4.8	101.5 ± 2.5	−75.6 ± 1.2	35–37°C pp, LJP: nk	Ma et al., 2011
iPSC-CM V	28 ± 5	495 ± 36	9.5 ± 1.8	109 ± 3	−57 ± 1	32°C, wc LJP: nk	Itzhaki et al., 2011a
iPSC-CM^*^ ns	60	173.5 ± 12.2	115.7 ± 18.4	106 ± 3.2	−72.4 ± 0.9	37°C, pp LJP: corr	Davis et al., 2012
hESC-CM V	47.1 ± 23.3	247.2 ± 66.7	13.2 ± 6.2	85.4 ± 9.3	−53.9 ± 8.6	37°C, me LJP: nk	He et al., 2003
hESC-CM V		285.8 ± 52.6	11.4 ± 2.8	86.8 ± 52.6	−62.3 ± 8.6	rt, wc LJP: nk	Zhang et al., 2011
VM^*^	50	213 ± 7	215 ± 33	106.7 ± 1.4	−81.8 ± 3.3	37°C, wc LJP: nk	Magyar et al., 2000
VM endo^*^	60	330 ± 16	234 ± 28	105 ± 2	−87.1 ± 1	37°C, wc LJP: nk	Drouin et al., 1998
VM epi^*^	60	351 ± 14	228 ± 11	104 ± 2	−86 ± 1	37°C, wc LJP: nk	Drouin et al., 1998

iPS-CM V, iPS-CM of ventricular-like phenotype; iPS-CM ns, not specified to action potential type; VM, native human ventricular myocyte; endo, endocardial; epi, epicardial; BPM, beats per minute; APD_90_, action potential duration at 90% of repolarization; dV/dt_max_, maximal upstroke velocity; APA, action potential amplitude; MDP, maximum diastolic potential; RMP, resting membrane potential;

* , non spontaneous beating cells;

hESC-CM V, hESC-CM of ventricular-like phenotype; me, micro electrode; wc, whole cell patch-clamp configuration; ljp: corr, corrected for liquid junction potential; ljp:nk, not known if corrected for liquid junction potential; rt, room temperature.

### Membrane currents

**Figure 1:**
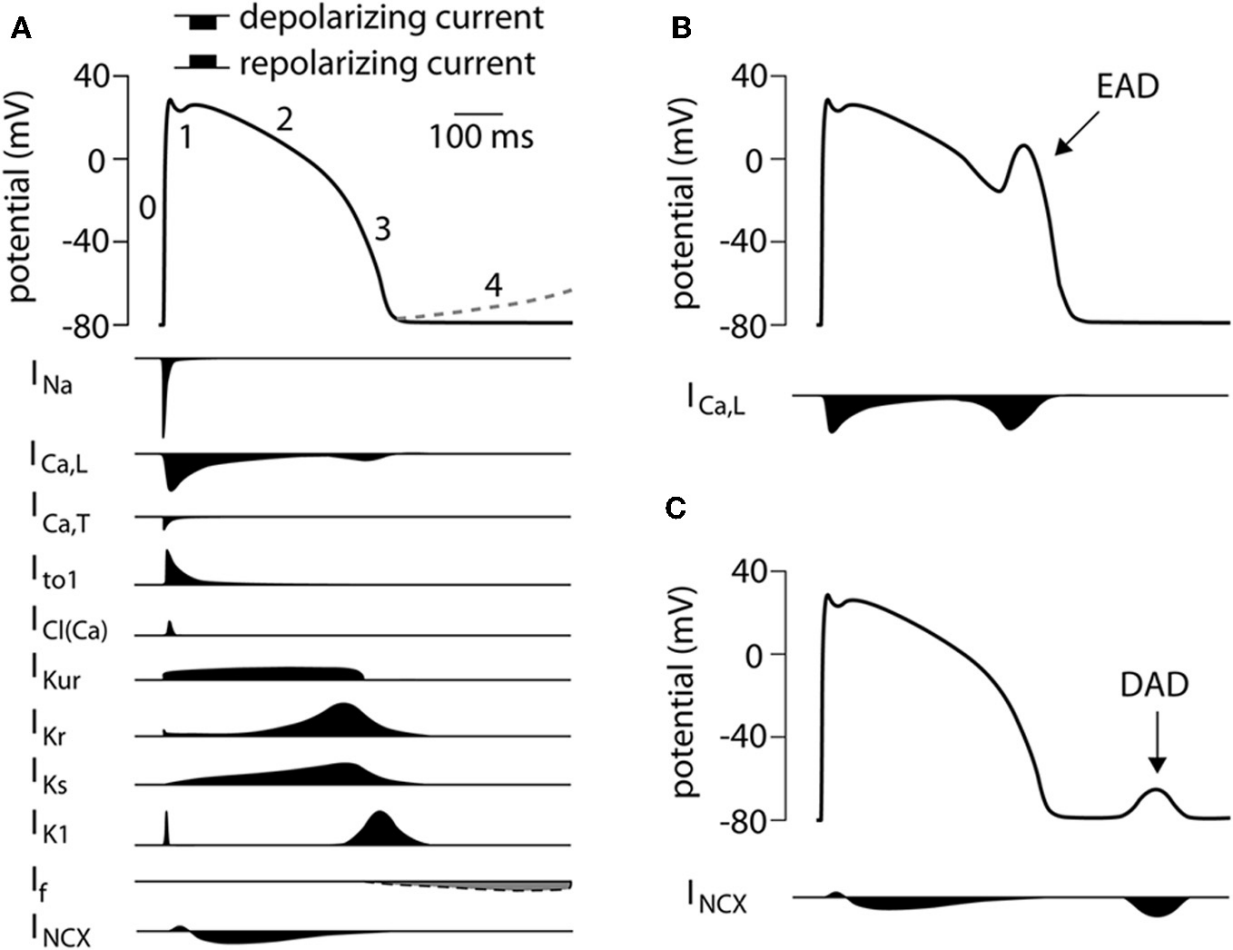
**(A)** Schematic representation of a human ventricular action potential (top panel). Numbers denote the different phases of the ventricular action potential. The dashed line represents phase 4 depolarization normally present in cells from the conduction system and not in ventricular CMs. Underlying ionic membrane currents and their schematic time course are depicted below. **(B)** Schematic representation of an early afterdepolarization (EAD) and its underlying mechanism. **(C)** Schematic representation of a delayed afterdepolarization (DAD) and its underlying mechanism. I_Na_, Na^+^ current; I_Ca,L_, L-type Ca^2+^ current; I_Ca,T_, T-type Ca^2+^ current; I_to1_, transient outward current type 1; I_Cl(Ca)_, Ca^2+^ activated Cl^−^ current, also called I_to2_; I_Kur_, ultra rapid component of the delayed rectifier K^+^ current, I_Kr_, rapid component of the delayed rectifier K^+^ current; I_Ks_, slow component of the delayed rectifier K^+^ current; I_K1_, inward rectifier K^+^ current; I_f_, funny current; I_NCX_, Na^+^/Ca^2+^ exchange current.

The shape of the AP is the result of the various inwardly and outwardly directed ion currents present in the CM. A schematic overview of the different ionic membrane currents underlying the ventricular AP and their course is depicted in Figure 1. Because of the clear differences in AP shape between native CMs and hiPSC-CMs, one can assume that differences exist in the content and function of the various cardiac ion channels between the two cell types. Thus, before hiPSC-CMs can be used as a cell model in the study of cardiac arrhythmia syndrome, it is important to carry out a detailed comparison between the cardiac ion currents in hiPSC-CMs with those in native CMs. In the description of the cardiac ion currents below, a comparison between hiPSC-CMs displaying ventricular-like APs and healthy native human ventricular CMs is made, unless stated otherwise.

#### Sodium current

The cardiac Na^+^ current (I_Na_) is responsible for the AP upstroke in ventricular CMs [see (Berecki et al., 2010), and primary references cited therein]. Mutations in the genes encoding the α- and β-subunits of the cardiac Na^+^ channel can alter the kinetics and availability of the cardiac Na^+^ current (Remme et al., 2008). As stated before, the upstroke velocity in hiPSC-CM APs is extremely low compared to the AP upstroke of freshly isolated human ventricular CMs. In hiPSC-CM, I_Na_ was studied in detail in two reports (Ma et al., 2011; Davis et al., 2012). Ma et al. (2011) report a half-maximal potential (V_1/2_) of activation and inactivation of –34.1 and –96.1 mV, respectively. Davis et al. (2012) report a V_1/2_ of activation of ~42 mV. The findings of these studies are consistent with values reported for native human ventricular CMs (Sakakibara et al., 1993) (Table 2). The low temperature and reduced Na^+^ concentration used to study the maximal peak I_Na_ in native human ventricular CM (Sakakibara et al., 1993) prevents comparison with the maximal peak I_Na_ measured in hiPSC-CM (Ma et al., 2011; Davis et al., 2012). Other I_Na_ characteristics, such as recovery from inactivation and slow inactivation have not been reported to date. In the presence of the Na^+^ channel blocker tetrodotoxin (TTX) the upstroke of the AP in hiPSC-CMs is delayed and the *dV*/*dt*_max_ is reduced (Ma et al., 2011). In hESC-derived CMs (hESC-CM) the Na^+^ channel blocker lidocaine also reduced the spontaneous beating rate (Kuzmenkin et al., 2009). Whether I_Na_ plays a role in spontaneous activity in hiPSC-CM is unknown. However, hiPSC-CMs have prominent Na^+^ currents with characteristics close to that of native human ventricular CMs, despite any information to compare maximal peak I_Na_, it seems that the low *dV*/*dt*_max_ of spontaneously active ventricular-like hiPSC-CM APs seems thus due to lower functional availability of Na^+^ channels (related to the relative positive value of the RMP) rather than differences in I_Na_ density.

**Table 2:** **Ion channel current and gating properties in hiPS-CM and human cardiomyocytes**.

	**Cell type**	**Peak current (pA/pF)**	**V_1/2_ act (mV)**	**k act**	**V_1/2_ inact (mV)**	**k inact**	**Exp. conditions**	**Reference**
I_Na_	iPS-CM	−216.7 ± 18.7	−34.1	5.9	−72.1	−5.7	35–37°C wc, LJP: nk	Ma et al., 2011
	iPS-CM	~280 (Figure 8C)	~ −42 (Figure 8D)				37°C, pp LJP: nc	Davis et al., 2012
	hESC-CM	−72 ± 21	−34	5.7	−78	−4.6	20°C, wc LJP: nk	Jonsson et al., 2012
	VM	−20.2 ± 2.2	−42.4 ± 3.0	6.3 ± 0.5	−100 ± 2.1	−6.3 ± 0.5	17°C, wc LJP: nk	Sakakibara et al., 1993
I_Ca,L_	iPS-CM	−17.1 ± 1.7	−14.9	6.6	−29.1	−4.9	35–37°C wc, LJP: nk	Ma et al., 2011
	iPS-CM	−3.3	~ −45 (Figure 2E)				25°C, wc LJP: nk	Yazawa et al., 2011
	hESC-CM	−4.3 ± 0.6	−12	5.5			37°C, wc LJP: nk	Jonsson et al., 2012
	VM	−10.2 ± 0.6	−4.7 ± 0.7	3.7 ± 0.3	−19.3 ± 1.2	−3.45 ± 0.6	37°C, wc LJP: nk	Magyar et al., 2000
	VM	−3.8 ± 0.5	−4.2 ± 0.8	7 ± 0.3	−23.5 ± 1.4	−5.5 ± 1.4	21–23°C wc, LJP: nk	Mewes and Ravens, 1994
	AM	−2.2 ± 0.3	−12.1 ± 1.3	5.8 ± 0.4	−26.8 ± 1.3	−5.7 ± 0.2	21–23°C wc, LJP: nk	Mewes and Ravens, 1994
I_to1_	iPS-CM	~2.4 (Figure 6D)					35–37°C wc, LJP: nk	Ma et al., 2011
	iPS-CM	~30 (Suppl. Figure 10A)					35°C, wc LJP: nk	Moretti et al., 2010
	hESC-CM	6.0 ± 0.9					37°C, wc LJP: nk	Sartiani et al., 2007
	VM endo	~5 (Figure 2B)	29.1 ± 1.2	12.9 ± 1.1	−17.6 ± 1.0	−8.9 ± 0.9	35°C, wc LJP: nk	Nabäuer et al., 1996
	VM epi	~16 (Figure 2A)	32.0 ± 1.11	14.9 ± 0.8	−9.5 ± 0.38	−5.1 ± 0.4	35°C, wc LJP: nk	Nabäuer et al., 1996
	VM endo	2.3 ± 0.3	23.1 ± 4.2	12.9 ± 0.8	−25.3 ± 3.0	−4.7 ± 0.8	20–22°C wc, LJP: nk	Wettwer et al., 1994
	VM epi	7.9 ± 0.7	15.4 ± 0.7	9.7 ± 1.6	−31.9 ± 1.5	−4.6 ± 0.2	20–22°C wc, LJP: nk	Wettwer et al., 1994
I_Kr_	iPS-CM	0.95 ± 0.02	−22.7	4.9			35–37°C wc, LJP: nk	Ma et al., 2011
	iPS-CM	~1.9 (Figure 4A)					35°C, wc LJP: nk	Moretti et al., 2010
	iPS-CM	~1.9 (Figure 5C)					36°C, pp LJP: nk	Lahti et al., 2012
	iPS-CM	~0.55 (Figure 3D)					32°C, wc LJP: nk	Itzhaki et al., 2011a
	hESC-CM	−11.5 ± 1.8					37°C, wc LJP: nk	Jonsson et al., 2012
	VM	~0.25 (Figure 1C)	−5.74	5.63			37°C, wc LJP: nk	Iost et al., 1998
	VM	0.31 ± 0.02					37°C, wc LJP: nk	Magyar et al., 2000
I_Ks_	iPS-CM	0.31 ± 0.09					35–37°C wc, LJP: nk	Ma et al., 2011
	iPS-CM	~2.5 (Figure 4A)					35°C, wc LJP: nk	Moretti et al., 2010
	hESC-CM	0.65 ± 0.12					37°C, wc LJP: nk	Jonsson et al., 2012
	VM	0.18					37°C, wc LJP: nk	Virag et al., 2001
I_K1_	iPS-CM	~ −3.8 (Figure 6C)					35–37°C wc, LJP: nk	Ma et al., 2011
	hESC-CM	−2.67 ± 0.3					37°C, wc LJP: nk	Jonsson et al., 2012
	VM	~ −10 (Figure 3B)					37°C, wc LJP: nk	Magyar et al., 2000
I_f_	iPS-CM	−4.1 ± 0.3	−84.6	8.8			35–37°C wc, LJP: nk	Ma et al., 2011
	hESC-CM	−10 ± 1.1	−74	4.5			37°C, wc LJP: nk	Jonsson et al., 2012
	VM	−1.18 ± 0.21	−111 ± 0.06				22°C, wc LJP: nk	Hoppe et al., 1998
	SAN	~ −8.0 (Figure 3B)	−96.9 ± 2.7	8.8 ± 0.5			36°C, wc LJP: corr	Verkerk et al., 2007

VM, native human ventricular myocyte; AM, native human atrial myocyte; endo, endocardial; epi, epicardial; SAN; native sinoatrial node myocyte; ~, estimated from figure. I_Na_, Na^+^ current; I_Ca,L_, L-type Ca^2+^ current; I_to1_, Transient outward current type 1; I_Kr_, rapid compontent of the delayed rectifier K^+^ current; I_Ks_, slow component of the delayed rectifier K^+^ current; I_K1_, inward rectifier K^+^ current; I_f_, funny current; V_1/2_ act, potential of half maximal activation; k act, slope factor of activation curve; V_1/2_ inact, potential of half maximal inactivation; k inact, slope factor of inactivation curve hESC-CM; V, hESC-CM of ventricular-like phenotype; wc, whole cell patch-clamp configuration; ljp: corr, corrected for liquid junction potential; ljp: nk, not known if corrected for liquid junction potential, ljp: nc, not corrected for liquid junction potential.

#### Calcium current

Two types of Ca^2+^ current exist in the mammalian heart, i.e., the L-type (I_Ca,L_) and T-type (I_Ca,T_) Ca^2+^ current [for review, see (Nerbonne and Kass, 2005)]. Patch clamp studies have demonstrated the presence of the I_Ca,L_ in hiPSC-CM with a V_1/2_ of activation and inactivation of –15 and –29 mV, respectively (Ma et al., 2011). These values are more comparable to those found in native atrial CMs (–12 and –27 mV, for V_1/2_ of activation and inactivation, respectively) (Mewes and Ravens, 1994) than native ventricular myocytes (–4.2 and –4.7 mV and –23.5 and –19.3 mV) (Mewes and Ravens, 1994; Magyar et al., 2000). The maximal peak I_Ca,L_ in hiPSC-CM reported by Ma et al. (2011) is 16.4 pA/pF, which is much higher than the 3.3 pA/pF reported in hiPSC-CM by Yazawa et al. (2011). In native ventricular CMs the amplitudes of the maximal current density varies between 2.2 and 10.2 pA/pF (Mewes and Ravens, 1994; Magyar et al., 2000) The higher maximal peak I_Ca,L_ in the study of Ma et al. (2011) may be explained by the higher extracellular Ca^2+^ and higher temperatures used in their experiments. Blocking of the I_Ca,L_ with nifedipine results in shortening of the AP duration and field potential duration (FDP) with minimal effects on *dV*/*dt*_max_ (Itzhaki et al., 2011a; Ma et al., 2011). Long-term application of nifedipine resulted in cessation of beating in some EBs (Itzhaki et al., 2011a).

Functional presence of the I_Ca,T_ has not been reported in hiPSC-CM. Ma et al. (2011) did not find clear evidence for its presence. I_Ca,T_ is present in the human heart conduction system, where it plays a role in facilitation of pacemaker depolarization, but is not functionally present in healthy human native ventricular CMs (Ono and Iijima, 2010). The T-type Ca^2+^ channels are re-expressed in atrial and ventricular CMs under pathological conditions such as cardiac hypertrophy and heart failure (Ono and Iijima, 2010).

#### Transient outward current

Two transient outward current components are found in native mammalian cardiac cells, one carried by K^+^(I_to1_), the other by Cl^−^ ions (I_to2_) [for review, see (Nerbonne and Kass, 2005)]. While it is not yet known whether I_to2_ is present in hiPSC-CMs, native human ventricular CMs are known to lack I_to2_ (Verkerk et al., 2001). On the otherhand, I_to1_ has been found in hiPSC-CMs (Moretti et al., 2010; Ma et al., 2011), but data about gating properties are not known. Reported peak current densities of I_to1_ in hiPSC-CM display large variation, namely between 2.4 (Ma et al., 2011) and 30 pA/pF (Moretti et al., 2010), both at 60 mV. Values reported for native ventricular CMs vary between 2.3 and 16 pA/pF (Wettwer et al., 1994; Nabäuer et al., 1996). Although an exact comparison of I_to1_ density between hiPSC-CM and native human CMs is hampered by differences in experimental conditions, I_to1_ current density also depends on the site from which the native ventricular CMs are isolated, with larger current densities reported in human subepicardial ventricular myocytes compared to endocardial ventricular CMs (Beuckelmann et al., 1993; Wettwer et al., 1994).

Studies of I_to1_ block on hiPSC-CM APs have not yet been performed. However, due to the depolarized MDP values in hiPSC-CM, I_to1_ function may be limited because most channels will be inactivated (Varro and Papp, 1992). Further studies are required to address the function of I_to1_ in hiPSC-CM in detail.

#### The delayed rectifier potassium current

In the mammalian heart, the delayed rectifier K^+^ current (I_K_) is composed of three different components: the ultrarapid (I_Kur_), the rapid (I_Kr_), and the slow (I_Ks_) components [for review, see (Nerbonne and Kass, 2005)]. To our knowledge, studies to elucidate the presence and function of I_Kur_ in hiPSC-CMs are lacking.

I_Kr_ has been reported in hiPSC-CM with a maximal I_Kr_ density varying between 0.55 and 1.9 pA/pF (Moretti et al., 2010; Itzhaki et al., 2011a; Ma et al., 2011; Lahti et al., 2012), values comparable to those in native human CMs where I_Kr_ densities are between 0.25 and 0.6 pA/pF (Iost et al., 1998; Magyar et al., 2000; Jost et al., 2009). V_1/2_ of activation is –22.7 mV (Ma et al., 2011) in hiPSC-CM and –5.74 mV in native ventricular CMs (Iost et al., 1998) and –14 mV in atrial CMs (Wang et al., 1994). In hiPSC-CMs, blockade of I_Kr_ by E4031 resulted in a significant AP prolongation In addition, E4031 induced early afterdepolarizations (EADs) (Itzhaki et al., 2011a; Ma et al., 2011; Matsa et al., 2011; Lahti et al., 2012), that is afterdepolarization during the AP (Figure 1B) due to the reactivation of I_Ca,L_ (Verkerk et al., 2000b). Thus, I_Kr_ plays a prominent role in the repolarization phase of hiPSC-CM APs. Interestingly, in hESC-CM E4031 reduced the beating rate (Kuzmenkin et al., 2009) suggesting that I_Kr_ also plays a role in spontaneous activity.

The presence of I_Ks_ in hiPSC-CMs has been reported in two studies. Ma et al. (2011) found I_Ks_ in 5 out of 16 cells studied, and when present the average I_Ks_ density was 0.31 pA/pF. In contrast, Moretti et al. (2010) measured I_Ks_ in all studied cells and the average density was around 2.5 pA/pF [estimated from Figure 4A (Moretti et al., 2010)]. In native left ventricular human CMs, Virag et al. (2001) identified I_Ks_ in 31 out of 58 cells and the maximal current density was approximately 0.18 pA/pF. In hiPSC-CMs, blockade of I_Ks_ by chromanol 293B results only in minimal prolongation of the AP (Ma et al., 2011). This is consistent with the relative small number of cells exhibiting I_Ks_ and the small I_Ks_ densities found in their study, but contrast with the effects of a loss-of-function I_Ks_ mutation which results in a prominent AP prolongation (see paragraph “LQT1”). A study by Wang et al. (2011) in hESC-CMs suggests altered expression of the β-subunit mink encoded by the *KCNE1* gene, as a mechanism for variable I_Ks_ function in the developing heart and in disease.

#### Inward rectifier current

In atrial and ventricular CMs, the inward rectifier K^+^ current (I_K1_) is an important contributor to the maintenance of the RMP and contributes to the terminal phase of repolarization (Dhamoon and Jalife, 2005). I_K1_ was found to be present in hiPSC-CM (Ma et al., 2011). I_K1_ density in hiPSC-CM is four times smaller than that reported in native ventricular CMs, 0.9 (Ma et al., 2011) and 3.6 pA/pF (Magyar et al., 2000), respectively. In hESC-CMs I_K1_ is significantly increased in longer cultured hiPSC-CMs, these cells also displayed a flattened diastolic depolarization rate and decreased spontaneous activity (Sartiani et al., 2007).

The small I_K1_ densities in hiPSC-CMs may explain the frequently observed spontaneous activity in these cells. However, whether all hiPSC-CMs have a low I_K1_ density or whether a bias is introduced by the selection of spontaneously active cells for patch-clamp needs to be elucidated.

#### The acetylcholine-activated K^+^ current

The acetylcholine-activated K^+^ current (I_K,ACh_) is involved in parasympathetic regulation of heart rate (Tamargo et al., 2004). I_K,ACh_ is to our knowledge not yet studied in hiPSC-CM. Studies addressing the presence of I_K,ACh_ in hiPSC-CM might be particularly important in modeling atrial arrhythmias, as blockers of I_K,Ach_, which leave ventricular repolarization intact, are effective in the treatment of atrial fibrillation (Hashimoto et al., 2006).

#### The ATP-sensitive K^+^ current

The ATP-sensitive K^+^ current (I_K,ATP_) has not been studied in detail in hiPSC-CMs. However, the I_K,ATP_ channel openers nicorandil and pinacidil shorten the AP in hiPSC-CMs (Itzhaki et al., 2011a; Matsa et al., 2011), suggesting that I_K,ATP_ channels are present in these cells. Further studies are required to address the presence and function of I_K,ATP_ in hiPSC-CM in detail.

#### The hyperpolarization-activated “funny” current

The funny current (I_f_) is an inward current activating at hyperpolarized membrane potentials [for review, see (Verkerk et al., 2007)]. In human sinoatrial node cells, the current density of I_f_ at a membrane potential of –130 mV is reported to be 8 pA/pF (Verkerk et al., 2007). I_f_ is also described in human atrial CMs (El Chemaly et al., 2007) and in human ventricular CMs during heart failure [(Hoppe et al., 1998)]. The current densities reported, however, are much smaller, compared to human sinoatrial node cells. hiPSC-CMs also exhibit I_f_ (Ma et al., 2011) and the reported current density is 4.1 pA/pF (Ma et al., 2011). The relatively high I_f_ density in hiPSC-CMs compared to human ventricular CMs might be attributed to the fact that these cells express higher levels of the HCN isoforms (HCN1, 2, 4) as compared to adult human CMs. (Synnergren et al., 2012). In hiPSC-CMs, I_f_ starts to activate at potentials negative of –60 mV and has a V_1/2_ of activation of –84 mV (Ma et al., 2011) and may therefore have a role in spontaneous activity in these cells. hESC-CMs have comparable characteristics of I_f_, and in these cells blockade of I_f_ with zatebradine resulted in slowing of spontaneous activity due to a reduced diastolic depolarization rate (Sartiani et al., 2007).

#### Na^+^-Ca^2+^ exchange current

The Na^+^-Ca^2+^ exchange current (I_NCX_) is crucial for Ca^2+^ extrusion from the cell and plays a role in the electric activity of mammalian CMs (Sipido et al., 2007). While the functional properties of I_NCX_ have not been studied in hiPSC-CM, the presence of the Na^+^-Ca^2+^ exchanger in hiPSC-CM has been demonstrated at the level of the protein (Lee et al., 2011). I_NCX_ is present in hESC-CM and its density increases with maturation (Fu et al., 2010).

In human CMs, Ca^2+^ extrusion by the Na^+^/Ca^2+^ exchanger is the major mechanism to balance the Ca^2+^ influx through the I_Ca,L_ (Sipido et al., 2007). In addition, the Na^+^/Ca^2+^ exchanger has a function during depolarization where it contributes in its reverse mode (Ca^2+^ influx) to the total amount of Ca^2+^ influx. The amplitude of the I_NCX_ depends on the membrane potential and the intracellular levels of Na^+^ ([Na^+^]_i_) and Ca^+^ ([Ca^2+^]_i_) (Sipido et al., 2007). Altered [Na^+^]_i_ and/or [Ca^2+^]_i_ will lead to altered I_NCX_ and can cause cardiac arrhythmias due to spontaneous Ca^2+^ releases from the SR. Studying the I_NCX_ in hiPSC-CM might be of particular interest in cardiac arrhythmia models of CPVT and LQT3. CPVT is associated with mutations in the RyR2 which can lead to increased [Ca^2+^]_i_ due to altered gating properties of the RyR2 receptor. In LQT3 syndrome there is an increased persistent I_Na_ (Remme et al., 2006), which might lead to elevated levels of [Na^+^]_i_.

### Excitation-contraction coupling

hiPSC-CMs display clearly visible contractions. In native adult CMs, a small influx of Ca^2+^ through the L-type Ca^2+^ channels triggers a several-fold multiplied Ca^2+^ release from the sarcoplasmic reticulum (SR) via ryanodine receptors (RyRs). This phenomenon is referred to as “Ca^2+−^induced Ca^2+^ release” (CICR) (Lee et al., 2011). CICR is the key mechanism underlying excitation-contraction coupling. The key Ca^2+^ handling proteins, RyR2, SR Ca^2+^-ATPase (SERCA), junctin (Jun), triadin (TRDN), Na^+^/Ca^2+^ exchanger (NCX), calsequestrin (CASQ2), L-type Ca^2+^ channel (Ca_v_1.2), inositol-1,4,5-trisphosphate receptor (IP3R2) and PLN are expressed in hiPSC-CM (Itzhaki et al., 2011b; Lee et al., 2011). Spontaneous rhythmic Ca^2+^ transients are present in hiPSC-CM, and blocking of the I_Ca,L_ by nifedipine, abolishes Ca^2+^ transients (Itzhaki et al., 2011b). The presence of functional SR and RyRs was proven by application of caffeine, which induced a large Ca^2+^ transient (Itzhaki et al., 2011b; Lee et al., 2011), consistent with findings in human ventricular CMs (Piacentino et al., 2003). In addition, ryanodine caused a reduction in the amplitude of the Ca^2+^ transient (Itzhaki et al., 2011b; Lee et al., 2011). The pattern of Ca^2+^ transient in hiPSC-CM was studied by transverse line-scan images and revealed a U-shape Ca^2+^ wavefront (the rise of Ca^2+^ in the periphery is faster than in the center of the cell), which is typical for t-tubule deficient cells (Lee et al., 2011). This suggests that hiPSC-CMs lack t-tubuli, an observation which is in line with that of Novak and co-workers (Novak et al., 2012) who did not find t-tubuli with transmission electron microscopy. This would mean that hiPSC-CMs likely have poor coupling between Ca^2+^ influx through L-type Ca^2+^ channels and Ca^2+^ release from the SR through RyRs.

## Human iPSC-CM models for inherited cardiac arrhythmias

### hiPSC-CM models for LQTS

#### LQT1

Moretti and co-workers (Moretti et al., 2010) were the first to publish on a hiPSC-CM model for a primarily electrical disease, namely LQT1. LQT1 is a repolarization disorder identified by a prolongation of the QT interval on the ECG due to mutations in the *KCNQ1* gene, encoding the α subunit of the K^+^ channel responsible of I_Ks_ (Wilde and Bezzina, 2005). They investigators obtained fibroblasts from two, so far, asymptomatic patients, with the *KCNQ*1-G569A mutation and two healthy controls (Moretti et al., 2010). These fibroblasts were infected with retroviruses encoding OCT3/4, SOX2, KLF4, and c-MYC; hiPSC-CMs were differentiated as EBs. In this study AP characteristics and K^+^ currents were investigated in spontaneously beating cells. Three different types of AP s were distinguished, that were designated as ventricular-, atrial-, and nodal-like. These investigators also correlated these characteristics with gene-expression analysis of specific myocyte-lineage markers (MLC2v, MLC2a, and HCN4 for ventricular-, atrial- and nodal-like cells, respectively). The delayed rectifier currents were studied in ventricular-like myocytes. In hiPSC-CMs derived from the LQT1 patient (LQT1-iPSC-CM), I_Ks_ peak and tail current densities were reduced by approximately 75%, and I_Kr_ conductance was unaffected. APs of atrial-like and ventricular-like hiPSC-CMs were significantly prolonged in LQT1-iPSC-CMs compared to control (WT-iPSC-CMs). Adaptation of the AP duration to higher pacing frequencies and the response to isoproterenol were impaired in LQT- iPSC-CM. EADs were elicited in response to isoproterenol (a β-adrenergic agonist) in 6 out of 9 LQT1-iPSC-CM and never in WT-iPSC-CM. When propranolol (a non-selective β-blocker) was applied the effect of isoproterenol was blunted. These data are in line with observations in LQT1 patients as these patients suffer from arrhythmias during increased heart rates caused by emotional stress or exercise. The data is also in line with the beneficial effects of β-blockers in suppressing arrythmias in these patients (Ruan et al., 2008). Immunocytochemistry revealed that the *KCNQ*1-G569A mutation leads to impaired trafficking and localization of the mutant channels.

#### LQT2

Three groups have published on hiPSC-CM models of LQT2 (Itzhaki et al., 2011a,b; Matsa et al., 2011; Lahti et al., 2012). LQT2 is a repolarization disorder caused by mutations in *KCNH2*, encoding I_Kr_ channels (Wilde and Bezzina, 2005).

Itzhaki et al. (2011a) reported on a hiPSC-CMs model generated from dermal fibroblasts obtained from a 28-year-old woman with a diagnosis of familial LQT2 due to the *KCNH2*-A614V mutation. Clinical data of the patient were not shown. Fibroblasts were reprogrammed by retroviral infection with vectors encoding for SOX-2, KLF4, and OCT4. In this study EB formation was used for differentiation of the hiPSC into CMs. In this study APs were measured from spontaneously contracting clusters and the hiPSC-CMs were classified as nodal-, atrial-, and ventricular-like. Prolongation of repolarization and predisposition to the development of EADs was shown in cells with atrial- and ventricular-like APs. For voltage clamp experiments the spontaneously beating clusters were dissociated to single cells. Peak amplitudes of I_Kr_ were found to be significantly smaller in LQT2-iPSC-CM compared to WT-iPSC-CM. FDP corrected for variations in beating frequency were longer in LQT2-iPSC-CM compared to WT-iPSC-CM. When I_Kr_ was blocked by the hERG blocker E4021, the AP prolonged and EADs were seen in 66% of the cells studied. Furthermore, the effects of agents that may have a therapeutic effect in preventing arrhythmias were also studied. These agents include nifedipine, pinacidil, and ryanodine. Because Ca^2+^ influx through L-type Ca^2+^ channels contributes to AP duration and has a role in EAD formation, inhibition of I_Ca,L_ by nifedipine was proposed to be anti-arrhythmic. Another anti-arrhythmic strategy proposed was to augment the repolarization currents, by the I_K,ATP_ channel opener pinacidil. Both interventions resulted in AP shortening and abolished propensity to EADs. This study also demonstrated that ranolazine, a blocker of the persistent I_Na_, did not shorten the AP duration, but prevented EADs.

Matsa et al. (2011) generated hiPSC-CMs of a symptomatic and an asymptomatic carrier of the G1681A mutation in *KCNH2*. The symptomatic patient, female with a QTc interval of up to 571 ms, experienced 11 episodes of syncope in 12 months. As is typical for LQT2, episodes occurred at arousal from sleep and not during competitive sports. Her mother was the asymptomatic individual studied; although her QTc-interval was prolonged she had not experienced any symptoms. The hiPSC-CMs were derived from punch biopsies of skin, which were reprogrammed by lentiviral delivery of OCT4, SOX2 NANOG, and LIN28; EB formation was used for differentiation of cells into CMs. I_Kr_ current characteristics were not studied. The derived hiPSC-CMs showed APs which were categorized as ventricular-, atrial- and pacemaker-like. Ventricular- and atrial-like APs from the symotomatic patient and her mother showed increased durations compared to APs of the genetically unrelated control; AP duration was less in the maternal hiPSC-CMs compared to those of the patient. Application of isoprenaline resulted in 25% of LQT2-iPSC-CMs in electrophysiological abnormalities, including EADs. Isoprenaline-induced arrhythmias were ameliorated by nadolol or propanolol, non-selective β-blockers. This is in line with the clinical picture in LQT2 as these patients experience arrhythmias due to increased heart rates caused by emotional and similar stress, mainly auditory stimulation and arousal from sleep. As for LQT1, LQT2 patients are often treated with β-blockers for prevention of cardiac events (Ruan et al., 2008). I_Kr_ blockade by E4031 resulted in prolongation of the AP duration and EADs in 30% of the LQT2-iPSC-CMs, but never in control cells. Nicorandil, an I_K,ATP_ channel opener, and PD-118057, an I_Kr_ channel enhancer, shortened the AP in LQT2-iPSC-CM, showing that potassium channel activators can normalize the prolonged repolarization in LQT2.

Lahti et al. (2012) derived hiPSC-CMs from an asymptomatic carrier of the R176W mutation in the *KCNH2*. This mutation is one of the four founder mutations of LQTS cases in Finland, and present in one in 400 Finns (Marjamaa et al., 2009). The QTc intervals of patients carrying the R176W mutation range from 386 to 569 ms, with a mean of 448 ms (Fodstad et al., 2006). In the study of Lahti et al. (2012), fibroblasts were infected with lentivirus followed by retroviruses encoding for OCT4, SOX2, KLF4, and MYC to generate iPSC-CMs. CM differentiation was achieved by co-culturing hiPSC with END-2 cells. APs were divided into two types, atrial- and ventricular-like APs. Only the ventricular-like APs showed significantly increased APD_90_. The AP frequency had a tendency toward slower frequencies in LQT2-iPSC-CM. EADs were present in 1 of 20 LQT2 iPSC-CMs and were never observed in WT-iPSC-CMs. I_Kr_ step and tail current densities were reduced by 40–46%. The APs of LQT2-iPSC-CMs had a significantly prolonged duration compared to WT AP, especially at low frequencies. The I_Kr_ blocker E4031 provoked EADs in WT-iPSC-CM and LQT2-iPSC-CM, with the effect on LQT2-iPSC-CMs being more pronounced. Sotalol, a non-selective β-blocker elicited EADs only in LQT2-iPSC-CM.

These three studies on LQT2 show that LQT-iPSC-CMs of symptomatic patients show a more severe cellular phenotype than those obtained from asymptomatic patients with the same mutation. However, assessing severity in the hiPSC-CMs system is challenging. For instance, blocking I_Kr_ by E-4031 in WT-iPSC-CMs leads to different findings in different studies. In the study of Matsa et al. (2011) no EADs were provoked by the application of E4031, whereas in the study of Ma et al. (2011) and Lahti et al. (2012) EADs could be provoked in >50% of WT-iPSC-CM. This might reflect diversity of hiPSC-CM lines. However, the differences between the outcomes of these studies might also be caused by the use of different concentrations E-4031. Ma et al. (2011) and Lahti et al. (2012) used a concentration of 100 nmol/l and 500 nmol/l, respectively. The concentration used by Matsa et al. (2011) is not known.

#### LQT3/conduction disease/BrS

LQT3 is a repolarization disorder caused by gain-of-function mutations in *SCN5A* encoding the cardiac Na^+^ channel. These mutations cause an increased persistent Na^+^ current which acts to prolong CM repolarization and increase AP duration (Wilde and Bezzina, 2005). On the otherhand, SCN5A mutations associated with loss of channel function cause conduction disease and Brugada Syndrome (BrS). The latter is an arrhythmia syndrome characterized by ST segment elevation in the right precordial leads of the EGC; SCN5A mutations account for around 20% of BrS cases (Wilde and Bezzina, 2005). Loss of Na^+^ channel function leads to a decreased peak I_Na_, which causes slowing of the upstroke velocity of the AP.

Recently, we have, generated an iPSC-CM model of a patient carrying the *SCN5A*-1795insD mutation (Davis et al., 2012). This mutation gives rise to a phenotype of LQT3 as well as BrS and conduction defects, caused by both gain- and loss-of-function effects on the cardiac Na^+^ channel, respectively (Remme et al., 2006, 2009). In this study we generated hiPSC-CMs by transducing fibroblasts with lentiviral vectors encoding OCT4, SOX2, KLF4 and C-MYC (Davis et al., 2012). Cardiac differentiation was induced by co-culture with END-2 cells. In line with the known effects of the mutation in a knock-in mouse model, and in line with the clinical presentation in mutation carriers, we observed a decrease in peak I_Na_ and an increase in persistent I_Na_ in the hiPSC-CMs with *SCN5A*-1795insD compared to genetically unrelated control. APs measured in non-spontaneously active hiPSC-CMs displayed a reduced upstroke velocity and a prolonged duration compared to those derived from a genetically unrelated control.

#### LQT8/Timothy syndrome

LQT8 and Timothy syndrome are caused by mutation in the *CACNA1C* gene encoding the L-type Ca^2+^ channel. Repolarization disease is only one facet of LQT8 as *CACNA1C* mutations also give rise to other features including syndactyly, heart malformations, and autism spectrum disorders (Yazawa et al., 2011). Yazawa et al. (2011) studied hiPSC-CMs of two patients with LQT8. Fibroblasts were isolated from skin biopsies and were reprogrammed using four retroviruses containing SOX2, OCT3/4, KLF4, and MYC. EBs were used in the generation of hiPSC-CMs. EBs from LQT8/Timothy syndrome hiPSC lines contracted at 30 bpm, whereas control hiPSC-line EBs contracted at a rate of 60 bpm. The LQT8-iPSC-CM showed delay in inactivation of I_Ca,L_ and abnormalities in intracellular Ca^2+^ handling, with larger and prolonged Ca^2+^ transients. Importantly, such aspects of I_Ca,L_ mutations can not be revealed when studying the mutation in a heterologous cell system. The APs of LQT8/Timothy syndrome ventricular-like hiPSC-CMs were three times longer than those of WT hiPSC-CMs. Roscovitine, a compound that increases the voltage-dependent inactivation of the voltage dependent Ca^2+^ channel, reverted the delayed inactivation and restored the irregular Ca^2+^ transients associated with LQT8/Timothy syndrome.

### Catecholaminergic polymorphic ventricular tachycardia

CPVT is characterized by cateholamine/stress-induced ventricular arrhythmias that can lead to sudden cardiac death in young individuals (Priori and Chen, 2011). CPVT is linked to mutations in the *RYR2* gene, encoding an intracellular Ca^2+^ release channel, and mutations in *CASQ2*, encoding a calcium binding protein in the SR which stores Ca^2^. RyR2 and CASQ2 play a role in Ca^2+^ cycling and contractile activity of the CM (Priori and Chen, 2011). To date, three groups have published a CPVT hiPSC model (Fatima et al., 2011; Jung et al., 2012; Novak et al., 2012).

Fatima et al. (2011) studied the F243I mutation in the *RYR2* gene. A skin biopsy of a patient with CPVT carrying the F243I mutation in the *RYR2* gene was taken and fibroblasts derived from this biopsy were infected with retroviruses encoding OCT3/4. SOX2, KLF4, and c-MYC. Cardiac differentiation was achieved by co-culturing with END-2 cells. APs were measured in spontaneously beating single hiPSC-CMs and were categorized as ventricular-, atrial-, and nodal-like APs. Isoproterenol was used to evoke the phenotype. In 22 out of 38 CPVT-iPSC-CMs isoprenaline resulted in a negative chronotropic response and 13 cells exhibited delayed afterdepolarizations (DADs), which are afterdepolarizations after and AP (Figure 1C) due to spontaneous SR Ca^2+^ release what activates I_NCX_ (Verkerk et al., 2000a). All control hiPSC-CM showed normal positive chronotropic response. Confocal fluorescence imaging revealed spontaneous local Ca^2+^ release events of higher amplitude and longer duration in CPVT-iPS-CMs. In addition, the CPVT-iPSC-CMs showed a decrease in I_Ca,L_ and Ca^2+^ transients in the presence of forskolin, an adenyl cyclase activator. As the authors state, this is likely due to the large and sustained rise of intracellular Ca^2+^ concentration.

Jung et al. (2012) studied the *RYR2* mutation S406L. Fibroblasts of the patient were transduced with a retroviral vector encoding SOX2, OCT4, KLF4, and c-MYC. To direct the hiPSCs to the cardiac lineage, EB differentiation was used. The CPVT-iPSC-CM showed elevated Ca^2+^ concentrations, a reduced SR Ca^2+^ content, and increased susceptibility to DADs under catecholaminergic stress induced by isoproterenol. Further the authors investigated the ability of dantrolene to rescue the disease phenotype. Dantrolene is a hydrantoin derivative and muscle relaxant, currently used as therapy in cases with malignant hyperthermia, a disorder caused by mutations in the skeletal ryanodine receptor (*RYR1*) (Kobayashi et al., 2009). Dantrolene, restored normal Ca^2+^ spark properties and the arrhythmogenic phenotype.

Novak et al. (2012) studied the effect of the autosomal recessive missense mutation D307H in the *CASQ2* gene. Dermal fibroblasts of two mutation carriers were transduced with a single lentiviral vector containing OCT4, SOX2, KLF4, and c-MYC. Differentiation toward hiPSC-CMs was achieved by EB formation. Spontaneous beating rate of differentiated EBs was significantly lower in CPVT-iPSC-CMs (~26 bpm) compared to control iPSC-CMs (~39 bpm). Isoproterenol induced DADs, oscillatory arrhythmic prepotentials (diastolic voltage oscillation, which appear during the late diastolic depolarization) and increased [Ca^2+^]_i_.

## Conclusions and future perspectives

The hiPSC-CM models described in this review show that it is possible to recapitulate *in vitro* in the hiPSC-CM system the disease phenotype of patients with Mendelian cardiac rhythm disorders. Furthermore, different studies have shown that LQT-iPSC-CMs of symptomatic patients show a more severe cellular phenotype than those obtained from asymptomatic patients with the same mutation (Itzhaki et al., 2011a; Matsa et al., 2011; Lahti et al., 2012). Moreover, hiPSC-CMs can recapitulate a phenotype that cannot be shown in a heterologous expression system (Lahti et al., 2012). For example, Lahti et al. (2012) reported a decrease of ~43% in I_Kr_ density in LQT2- iPSC-CMs with the R176W mutation, which was not revealed in a heterologous expression system. This might reflect, amongst others, differences in cellular environment between the two cell systems or may be due to the effect of high transgene expression as a consequence of the use of a strong promoter in the heterologous expression system. A significant advantage of the hiPSC-CM system is that in this system, in contrast to heterologous expression systems, it also possible to study the effects on the AP and Ca^2+^ cycling.

The responses to some pharmacological agents are studied in hiPSC-CMs. The results are in line with what is seen in patients, healthy human beings, and adult CM. For example β-adrenergic stimulation with isoproterenol leads to a positive chronotropic effect and AP shorterning (Zhang et al., 2009; Moretti et al., 2010) and application of β-blockers blunt the effect of isoproterenol (Moretti et al., 2010). Also the AP shortening effects of I_K,ATP_ openers pinacidil and nicorandil and the I_Ca_ channel blocker nifedipine was captured in hiPSC-CM.

Functional I_Na_ (Ma et al., 2011; Davis et al., 2012), I_Ca,L_(Itzhaki et al., 2011a; Ma et al., 2011; Yazawa et al., 2011), I_Kr_ (Itzhaki et al., 2011a; Ma et al., 2011; Matsa et al., 2011; Lahti et al., 2012) and I_Ks_(Ma et al., 2011) have been demonstrated in hIPSC-CMs and mutations affecting these channels as well as pharmacological ion channel blockade were shown to impact on the AP. While the functional presence of SR, RyRs and the Ca^2+^ binding protein CASQ2was demonstrated (Itzhaki et al., 2011b; Lee et al., 2011; Novak et al., 2012), studies have shown that the coupling between Ca^2+^ influx through L-type Ca^2+^ channels and Ca^2+^ release from the SR through RyRs is poor as a consequence of the lack of t-tubuli in hiPSC-CMS. Thus, the use of hiPSC-CMs to study certain cardiac arrhythmia syndromes, such as CPVT and LQT8, caused by mutations in one of the Ca^2+^ handling proteins is limited to the study of the biophysical properties of the affected protein. While I_to1_, I_K1_, and I_f_ are present in hiPSC-CMs (Ma et al., 2011), their contribution to electrical activity in these cells has not been proven by pharmacological blockade or through the effect of mutations in the respective genes. Openers of I_K,ATP_ shorten the AP (Itzhaki et al., 2011a; Matsa et al., 2011), so functional presence can be presumed but needs to be studied in more detail. The presence of I_NCX_ is also not studied in detail but its functional presence can also be presumed since intact Ca^2+^ handling has been demonstrated (Itzhaki et al., 2011b; Lee et al., 2011). Up till now there is no evidence for the functional presence or absence of I_K,ACh_.

A difficulty in the use of hiPSC-CM models is their immature electrophysiological phenotype, with depolarized MDP or RMP and slow AP upstroke velocities compared to native CMs. The MDP and upstroke velocity of hiPSC-CMs resemble more those of fetal CMs than adult CMs (Davis et al., 2011). Furthermore, most studies report that hiPSC-CM beat spontaneously, which is also characteristic of fetal CMs (Mummery et al., 2003). Of note, in our study on *quiescent* hiPSC-CMs we recorded a more-negative RMP and a faster AP upstroke velocity (Davis et al., 2012). Considering the importance of I_K1_ for setting the RMP, it is likely that the quiescent hiPSC-CMs have a larger I_K1_ than spontaneously beating hiPSC-CMs. It is not known whether quiescent cells were also present among the hiPSC-CM generated in studies in which spontaneously beating hiPSC-CMs were studied. Possibly, in these studies, the spontaneous activity was used as a tool to recognize CMs. A further consideration is that *cultured* adult CMs show a depolarized MDP and slower AP upstroke velocity compared to freshly isolated adult CMs. The depolarized MDP in cultured CMs is known to be caused by progressive decline in I_K1_ (Mitcheson et al., 1998). Another similarity between cultured ventricular myocytes and hiPSC-CMs is that different AP phenotypes are observed. In many hiPSC-CM studies different AP characteristics are observed and are classified as ventricular-like, atrial-like and nodal-like AP. Similarly, when ventricular CMs are cultured different AP phenotypes are observed after one day in culture, and more pronounced variability is evident after four days in culture (Mitcheson et al., 1998).

Another difficulty is the purity of the population of hiPSC-CMs acquired. With the current techniques it is not possible to acquire a pure population of CMs, the fraction of CMs obtained may vary from 1% to ~50% of the total cells (Dambrot et al., 2011). Moreover, as discussed above, the CMs that are generated are in fact a mixed population of CMs displaying different AP characteristics. One way in which this issue might be addressed is through the use of selectable markers driven by CM lineage-specific promoter elements. However, while this might be useful in selecting CMs as opposed to other cell types, more research is still required for the identification of promoter elements that may be used in selecting for specific CM types (e.g., ventricular versus atrial). Another solution might be found in chemical enhancement of cardiac differentiation. For instance, ascorbic acid enhances cardiac differentiation and minimizes the interline variance and facilitates the structural and functional maturation of hiPSC-CMs (Cao et al., 2012). From hESC-CM research we know that bone morphogenetic protein (BMP) signaling inhibition after mesodermal formation facilitates cardiac development. In this study, also a possibility for ventricular or atrial specific differentiation is shown (Zhang et al., 2011). Inhibition of retinoid (RA) signaling by noggin leads to CM specification into ventricular cells, whereas RA treatment leads to atrial specification (Zhang et al., 2011). Direction of differentiation to nodal like CMs can be achieved by activation of the Ca2^+^ activated potassium channels of small and intermediate conductance (SKCas) by 1-ethyl-2-benzimidazolinone (Kleger et al., 2010). Ventricular specification and electrophysiological maturation of hESC-CMs may also be promoted by microRNAs (miRs). MiR-499 was shown to promote ventricular specification; miR-1 facilitates electrophysiological maturation (Fu et al., 2011).

In summary, iPSC-CM models recapitulate the phenotype of patients with cardiac arrhythmia syndromes. However, the interpretation of electrophysiological data derived from these cells, should be done with caution, since hiPSC-CMs present immature phenotypes and do not recapitulate all the electrical characteristics of an adult CM. Thus, studies addressing the maturity and purity of the hiPSC-CMs acquired are needed, as well as studies to characterize the electrophysiological and pharmacological characteristcs in more detail. Because of this, there are still limitations for the use of this model system in studies on cardiac rhythm disorders, especially if the disease causing mutation is not known. So far, hiPSC-CMs have only been applied as models to the study of disorders for which mutations in particular genes have been identified. Future studies are likely to demonstrate the potential of these cell systems in pointing us to pathophysiological mechanisms for those cases for which no gene mutations are yet known. Another future application of hiPSC-CMs is the use in cardiac safety pharmacology and the development of new drugs. hiPSC-CMs provide researchers with CMs of human origin which are better-suited than CMs of animal origin or heterologous cell systems. Furthermore, hiPSC-CMs provide us with the opportunity to test drugs in disease-specific CMs instead of healthy CMs. Thus, in future studying hiPSC models might lead to novel insights in pathophysiology, improve understanding of genotype-phenotype relationships and could be used in the development and testing of pharmacological agents to treat human cardiac disease.

### Conflict of interest statement

The authors declare that the research was conducted in the absence of any commercial or financial relationships that could be construed as a potential conflict of interest.
